# A novel, sensitive and non‐destructive method for quantitative determination of lipid in live *Eriocheir sinensis* using low‐field ^1^H Nuclear magnetic resonance

**DOI:** 10.1002/fsn3.699

**Published:** 2018-09-27

**Authors:** Hongcai Zhang, Huaning Yu, Lingling Song, Shunsheng Chen

**Affiliations:** ^1^ College of Food Science and Technology Shanghai Ocean University Shanghai China; ^2^ Laboratory of Aquatic Products Quality and Safety Risk Assessment (Shanghai) at China Ministry of Agriculture Shanghai Ocean University Shanghai China; ^3^ State Key Laboratory of Dairy Biotechnology Shanghai Engineering Research Center of Dairy Biotechnology Dairy Research Institute Bright Dairy and Food Co., Ltd. Synergetic Innovation Center of Food Safety and Nutrition Shanghai China

**Keywords:** *Eriocheir sinensis*, lipid, non‐destructive analysis, nuclear magnetic resonance, Soxhlet extraction

## Abstract

In this study, lipid content of live *Eriocheir sinensis* has been quickly and accurately determined by low‐field ^1^H Nuclear magnetic resonance (LF‐^1^H NMR). The experimental parameters of LF‐^1^H NMR have been optimized and the validity of the established standard method has been confirmed with traditional Soxhlet extraction method. Results show that the lipid signal intensity is strongly correlated with its content and exhibits a good linear correlation (*Y* = 0.0376 + 4.899*X*,* R* ^2^ = 0.9999), thus demonstrating favorable accuracy and sensitivity for the quantitative determination of lipid content. In conclusion, the lipid content of live *E. Sinensis* can be directly obtained based on an established method, indicating a great application potential in food and other fields.

## 1 INTRODUCTION

Chinese mitten crab (*Eriocheir sinensis*) is an economically important type in China, and has existed for the last few decades. Due to their advanced hatchery techniques and high market demands, the total production of *E. sinensis* has reached up to 820,000 tons with a market value of 6 billion US dollars in 2015 (China Fishery Yearbook, 2015). The mixtures of liver and gonad tissues (crab roe) of *E. sinensis* are favorite raw food materials in Asian countries because of their excellent taste and high nutritional value (Jiang et al., 2009; Wang et al., 2016). Lipid mainly stored in liver and gonad tissues of *E. sinensis* play an important role in providing delicious flavor and abundant nutrients. Thus, the choice of an effective and efficient method to quantitatively calculate the lipid content of live *E. sinensis* is very critical and valuable.

To date, various analytical methods have been described for the determination of lipid content in food‐based samples, such as Near‐infrared (Chuang et al., 2016), Mid‐infrared (Georgouli, Del Rincon, & Koidis, 2017), Raman spectroscopy (Hall, Marshall, Gordon, & Killeen, 2016), and Soxhlet extraction method (Dinesha, Nidoni, Ramachandra, & Naik, 2016). A previous study has reported that the ovarian lipid concentration reached up to 19.1% in mature stage (stage IV) (Wen, Chen, Ai, Zhou, & Jiang, 2001) using the solvent extraction method. Although these approaches can provide both qualitative and quantitative results, they are time‐consuming, require expensive instruments, and are not suitable for rapid determination of lipid on a large scale.

Low‐field ^1^H Nuclear magnetic resonance (LF‐^1^H NMR) technique is a rapid, non‐destructive, highly reproducible, and sensitive technique (Hazlett et al., 1999; Seton, Hutchison, & Bussell, 1997), and has successfully been applied in quality control of food products such as porcine muscle (Qin, Xu, Zhou, & Wang, 2015; Shao, Deng, Jia, et al., 2016; Shao, Deng, Song, et al., 2016), pork (Shao, Deng, Jia, et al., 2016; Shao, Deng, Song, et al., 2016), salmon (da Silva Carneiro et al., 2016), crude lipid (Barbosa, Sad, Morgan, Figueiras, & Castro, 2016; Jia et al., 2016), egg (Zhao et al., 2016), milk (Salomonsen, Sejersen, Viereck, Ipsen, & Engelsen, 2007), honey (Ribeiro et al., 2014), and cod (Gudjónsdóttir, Arason, & Rustad, 2011). The LF‐^1^H NMR technique is often employed to investigate the water mobility and/or lipid content of foods because it can measure water or lipid proton relaxation. The proton relaxation is described in terms of relaxation time constants T_1_ (longitudinal) and T_2_ (transverse) because protons in different environments exhibit distinctive T_1_ or T_2_ relaxation properties. For the resolution of lipid hydrogen protons, T_2_ relaxation time is widely applied to collect more sample information, and has shown good consistency compared to traditional detection methods. However, little information is available for the determination of lipid content in live *E. sinensis*. To date, no study has been found to report the non‐destructive, quantitative determination of lipid content in live *E. sinensis* using LF‐^1^H NMR.

The objectives of this study are to establish a standard method for the quantitative determination of lipid content in live *E. sinensis*, and to verify the accuracy of LF‐^1^H NMR relative to the Soxhlet extraction method. The lipid content of live *E. sinensis* may directly be acquired once the detection method is established. This study provides a new procedure for the quick determination of lipid content of live *E. sinensis*.

## 2 MATERIALS AND METHODS

### 2.1 Materials and reagents

Eight female and male crabs (150 and 125 g, respectively) have been carefully picked and purchased from Chongming Aquaculture Culture Base in Shanghai. Polytetrafluoroethylene (PTFE) membrane has been obtained from Jiangsu Zhenjiang Hongke Bubber Co., Ltd, China. Anhydrous ether and ethanol and other reagents (analytical grade) have been purchased from Sinopharm Chemical Reagent Co., Ltd, China.

### 2.2 Optimization of LF‐^1^H NMR parameters

Prior to LF‐^1^H NMR optimization, experimental parameters of 2D ^1^H LF‐NMR to be considered include delaying time, waiting time (TW), and number of echo (NECH). The experimental parameters have been optimized using one 0.52 T MesoMR23‐60H‐I NMR system (Shanghai Niumag electronic technology Co., LTD, Shanghai, China). In LF‐^1^H NMR analysis, the 90° and 180° pulse lengths have been first adjusted using a free induction decay (FID) phase sequence before 2D ^1^H LF‐NMR measurement. Moreover, live *E. sinensis* are placed in a strong magnetic field, and a sequence of radio frequency pulses and magnetic field gradients have been used to localize the concentration and relaxation properties of hydrogen (H) protons. By choosing an appropriate pulse sequence, the signal intensity of samples can be reflected in terms of the T_1_ and T_2_ relaxation times obtained from Carr‐Purcell‐Meiboom‐Gill (CPMG) and inversion recovery (IR) sequences, respectively (Nakayama et al., 2015). Results of preliminary experiments showed that in terms of performance, T_2_ relaxation time was better than that of T_1_ relaxation because of “lipid‐water separation” studied in a previous report (Jia et al., 2016). Thus, T_2_ measurement has been utilized for further study.

### 2.3 Verification of lipid signal peak

To verify the signal peak location of lipid tissues in *E. Sinensis*, the liver and gonad tissues have been fetched and measured using LF‐^1^H NMR technique based on the optimized parameters.

### 2.4 Establishment of lipid detection method using LF‐^1^H NMR

**Figure 1:**
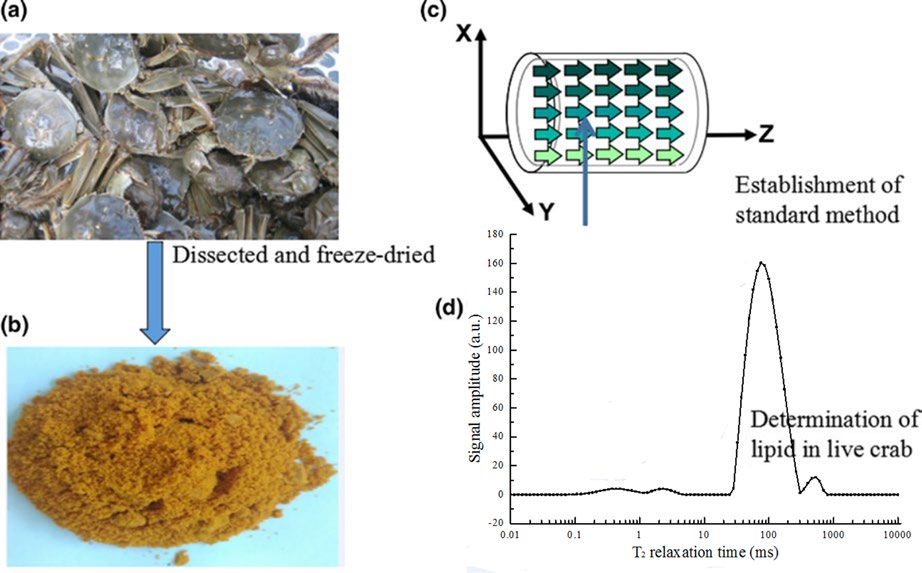
The quantitative determination of lipid content in live E. sinensis using low‐field 1H nuclear magnetic resonance (LF‐1H NMR).

The schematic diagram of detecting lipid content in crab roe using the LF‐^1^H NMR method is shown in Figure 1. The live *E. sinensis* have been weighed and dissected to extract the crab roe, and then freeze‐dried for further use. To put the crab roe into the standard LF‐^1^H NMR tube with length of 10 cm and diameter of 1.5 cm, the accurately weighed crab roe (0.5, 0.7, 1.0, 1.5, 2.0, 2.5, and 3.0 mg) are wrapped in a napkin (to eliminate signal interference) and loaded into test tubes. Each test tube is adjusted to the same height to eliminate signal interference and experimental error. The detections have been performed for establishing a linear relationship between signal intensity (peak position) of H protons and the weight of crab roe using the established LF‐^1^H NMR method. Hereafter, weighed *E. Sinensis* are wrapped with polyethylene film and put aside to eliminate the signal interference of water for 6 hr at ambient temperature. Then, live *E. Sinensis* are directly put into the 70 mm magnetic coil for the quantitative determination of lipid signal using T_2_ relaxation time, which has been calculated from the signal intensity (peak position) of H protons.

### 2.5 Validation analysis using Soxhlet extraction method

The extraction and detection of lipid have been investigated with minor modifications (Zhao & Zhang, 2014). The freeze‐dried crab roe is weighed and transferred to a filter paper, and then placed in the extractor of the Soxhlet apparatus (Model N‐1000S‐W; EYELA, Tokyo, Japan). The extractor is filled with 150 ml of anhydrous ether and heated under reflux for 8 hr at 78°C through a water bath. The solvent is removed at 60°C at 10 kPa using a rotary evaporator (Model SY2000; Shanghai Yarong Biochemistry Instrument Factory, China). The extraction is performed in triplicate and the total extraction yields are calculated as the mean value of the extracted lipid content in crab roe mass divided by the mass of raw crab used, on a dry weight basis.

### 2.6 Data analysis

The experimental results have been analyzed with a commercially available statistical package (SPSS Inc., Version 17.0, Chicago, IL, USA) based on the Least Significant Difference (LSD) test and Student–Newman–Keuls test (S‐N‐K) with a significance level of *p *<* *0.05. All measurements are performed in triplicate.

## 3 RESULTS AND DISCUSSION

### 3.1 Verification of lipid signal peak

**Figure 2:**
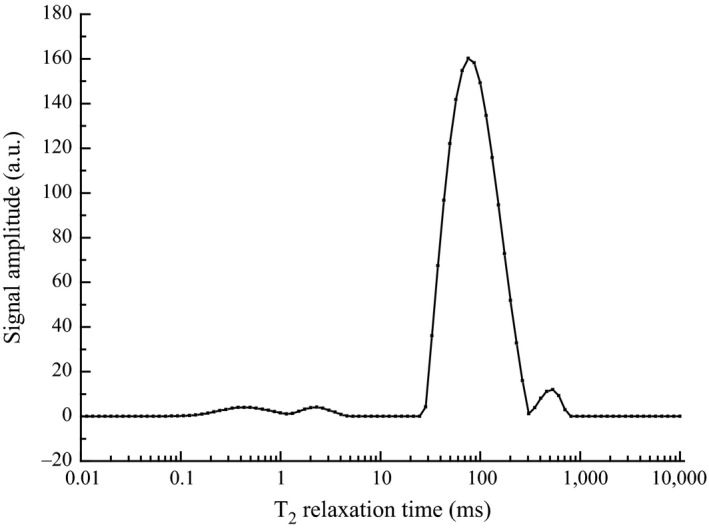
Verification of T_2_ relaxation time of *E. Sinensis*' lipid using low‐field‐^1^H Nuclear magnetic resonance (LF‐^1^H NMR)

Previous reports have showed that lipid peak may well be separated using T_2_ relaxation time (Hickey et al., 2006). T_2_ relaxation time of the lipid tissues in *E. sinensis* using LF‐^1^H NMR is shown in Figure 2. Two connected peaks in the range of 0–10 ms represent the bound water which integrates closely with polar groups on the surface of tissue protein molecules. The peak area of 28–300 ms is the largest, which indicates the lipid peak of liver and gonad tissues. The peak of 500–1,000 ms represents the signal peak of free water. The result shows that the appearance time of lipid peak between 28 and 300 ms is consistent with previous report (Shao, Deng, Jia, et al., 2016; Shao, Deng, Song, et al., 2016).

### 3.2 Establishment of a standard method for quantitative determination of lipid in live *E. sinensis*

**Figure 3:**
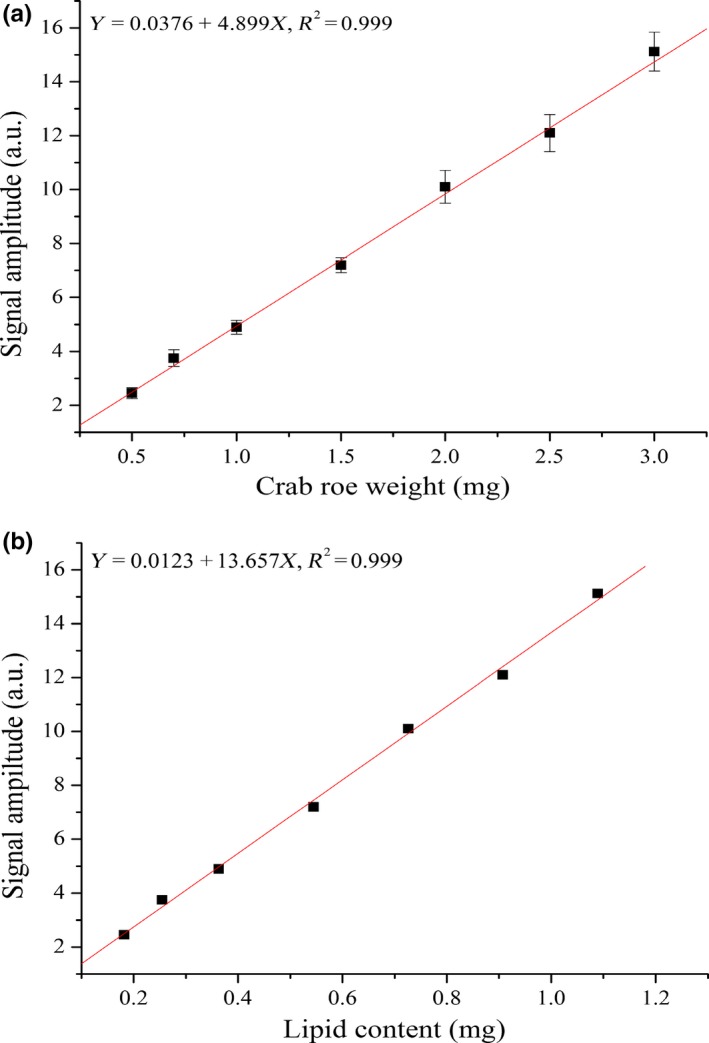
Calibration curve (a) for the determination of lipid content using low‐field‐^1^H Nuclear magnetic resonance (LF‐^1^H NMR) and the results' correlation (b) between Soxhlet extraction and LF‐^1^H NMR method

Prior to the establishment of the calibration curve for the quantitative determination of lipid using LF‐^1^H NMR, experimental parameters including delaying time, NECH, TW, and others have been optimized beforehand (Nakayama et al., 2015). The crab roe (0.5, 0.7, 1.0, 1.5, 2.0, 2.5, and 3.0 mg) is accurately weighed and the detections are performed according to the optimized experimental conditions described above. It has been observed that the signal intensity of H protons increases linearly with the weight of crab roe over the range from 0.5 to 3.0 mg (Figure 3a), thus showing high accuracy and sensitivity for the quantitative determination of lipid content, and the assay time is within 10 s for one sample. The calibration curve can be represented by the equation *Y* = 0.0376 + 4.899*X*,* R* ^2 ^= 0.9999. Therefore, the lipid content of live *E. sinensis* has been accurately and quantitatively determined via the established detection method.

### 3.3 Validation analysis of two kinds of methods

To compare the yields between the LF‐^1^H NMR method and the Soxhlet extraction method, the lipid content of live *E. sinensis* has been detected based on the previously established method (as shown in Table 1). The yield of lipid measured using the LF‐^1^H NMR method reaches 38 and 36% for the Soxhlet extraction method. Although the yields measured using the LF‐^1^H NMR method are slightly higher than that of the Soxhlet extraction method, there is no significant difference (*p* > 0.05). The reason is due to the fact that the loss of lipid using the Soxhlet extraction method is higher than that of the LF‐^1^H NMR method. Although the standard detection method needs to take a certain amount of time to work, the determination of the lipid content in live *E. sinensis* can be finished in just 10 s. The validation analysis suggests that the normalized lipid volume is strongly correlated with lipid content (Figure 3b), which exhibits a good linear correlation for male and female *E. sinensis* (*Y* = 0.0123 + 13.657*X*,* R* ^2 ^= 0.9999), the detection limit of lipid for crab roe being 0.15 mg. Therefore, LF‐^1^H NMR method is convenient and quick for the quantitative determination of lipid content on sites without complicated pretreatment of real samples. The established standard method can provide novel insights for the quantitative determination of lipid content in live *E. sinensis*, indicating its great application potential in quality evaluation and grading regulation.

**Table 1:** Comparative analysis of two methods for the determination of lipid content in live *E. Sinensis*

Treatment method	Crab weight* (g)	Signal amplitude (a.u.)	Crab roe (g)	Oil yields (%)
A_1_	52.18 ± 2.35^a^ †	103.4 ± 3.42^a^	–	37.95 ± 0.24^a^
A_2_	68.32 ± 3.86^b^	112.5 ± 5.83^b^	–	38.53 ± 0.15^a^
A_3_	89.34 ± 3.29^c^	126.8 ± 7.25^b^	–	38.53 ± 0.15^a^
B_1_	–	–	2.04 ± 0.24^a^	36.52 ± 1.18^a^
B_2_	–	–	2.35 ± 0.18^a^	36.28 ± 1.16^a^
B_3_	–	–	2.53 ± 0.36^a^	36.18 ± 1.29^a^

Notes

A and B represented the low‐field‐^1^H Nuclear magnetic resonance (LF‐^1^H NMR) and Soxhlet extraction method, respectively.

The same weight of *E. sinensis* is dissected to extract the crab roe after Non‐destructive detection using T_2_ measurements. ^†^In the same column, values with the same superscript letter (a–b) are not significantly different (*p *>* *0.05). Data are the means of three replications.

### 3.4 Comparison with Soxhlet extraction method

The comparison between both methods with respect to detection time, chemicals, waste, accuracy, and cost is shown in Table 2. The method proposed herein is fast and easy to perform. The procedures of LF‐^1^H NMR have been performed in approximately 10 s, whereas the Soxhlet extraction method takes more than 10 hr to finish. From the standpoint of cost, the new procedure corresponds to approximately 10% of that spent on the Soxhlet extraction method. Although the crab roe still need to be collected and freeze‐dried, the lipid content in live *E. sinensis* can directly be measured after the standard detection method is established. The equipment depreciation expense can effectively be reduced through random sampling in an aquaculture base. Comparing the use of chemicals and generation of waste, no chemicals have ever been used and no waste has ever been generated using the LF‐^1^H NMR method, but abundant anhydrous ether has been used, which has generated waste during the process for the Soxhlet extraction method. Moreover, the accuracy of the LF‐^1^H NMR method is significantly higher than that of the Soxhlet extraction method. Therefore, the technique based on LF‐^1^H NMR is a rapid, straightforward, and cost‐effective approach for the quantitative determination of lipid content in biological samples.

**Table 2:** Comparison between both methods with respect to detection time, chemicals, waste, accuracy, and cost

Parameters	LF‐^1^H NMR method	Soxhlet extraction method
Detection time	10 s	10 h
Chemicals	–	Anhydrous ether
Safety	High	Poor
Waste	–	Used anhydrous ether
Accuracy	High	Low
Cost	0.1 $	1‐3 $

Note

LF‐^1^H NMR: low‐field‐^1^H Nuclear magnetic resonance.

## 4 CONCLUSIONS

In this study, the standard method of detecting lipid content has been established by replacing the conventional method with LF‐^1^H NMR. The signal intensity of H protons to the content of lipid displays a good linear correlation over a range from 0.5 to 3.0 mg, demonstrating high accuracy and sensitivity for the quantitative determination of lipid content. The assay time is within 10 s. Therefore, the established approach can provide novel insights into calculating the lipid content of live *E. sinensis* using the LF‐^1^H NMR technique.

## CONFLICT OF INTEREST

The authors declare that they have no conflict of interest.

## ETHICAL STATEMENTS

Human and animal testing is not applicable in our study.
